# Effects of Nitrogen Addition on Leaf Functional Traits of Dominant Species in Bayanbulak Grassland, Xinjiang, China

**DOI:** 10.3390/plants14040597

**Published:** 2025-02-17

**Authors:** Xiaoyu Ding, Junjie Liu, Yao Wang, Juan Wang, Chao Liu, Mengtian Qin, Yujiao Xu, Yonggang Ma, Jianjun Yang, Zhonglin Xu

**Affiliations:** 1 College of Ecology and Environment, Xinjiang University, Urumqi 830017, China; 2 Technology Innovation Center for Ecological Monitoring and Restoration of Desert-Oasis, Ministry of Natural Resources Desert, Urumqi 830002, China; 3 Key Laboratory of Oasis Ecology, Ministry of Education, Xinjiang University, Urumqi 830017, China; 4 Institute of Desert Meteorology, China Meteorological Administration, Urumqi 830002, China; 5 College of Geography and Remote Sensing Sciences, Xinjiang University, Urumqi 830017, China

**Keywords:** nitrogen addition, alpine grassland, leaf functional traits, functional groups, structural equation modeling

## Abstract

Nitrogen inputs exert significant impacts on plant species composition and ecosystem stability within alpine grasslands. The exploration of leaf functional traits holds great potential in uncovering plants’ adaptive strategies and competitive edges, and is pivotal in comprehending the ramifications of nitrogen inputs on biodiversity. In this study, the Bayanbulak grassland was selected as the research subject to investigate the impact of nitrogen addition on leaf functional traits of different plant functional groups. Specifically, various gradients of nitrogen addition were established to observe changes in leaf dry matter content (LDMC) and leaf area (LA) among three distinct plant functional groups. Furthermore, structural equation modeling (SEM) was employed to analyze the pathways through which nitrogen addition influenced the LDMC of these plant functional groups. The results were as follows: (1) LA and leaf length (LL) of Poaceae changed significantly (*p* < 0.05) under different N addition gradients, and leaf nutrient contents of Poaceae, Rosaceae and Fabaceae showed significant changes under different N addition gradients. (2) Pearson correlation analyses showed that total nitrogen (TN), total carbon (TOC) and leaf width (LW) of Rosaceae leaves had a significant positive correlation, and the TOC and total phosphorus (TP) of Fabaceae leaves showed a significant negative correlation. (3) SEM of the three plant functional groups showed direct and indirect effects of N addition on leaf dry matter content of Poaceae and Rosaceae, and only indirect effects on Fabaceae.

## 1. Introduction

Plant functional traits can specifically reflect the adaptive strategies of plants to changes in the external environment, and can also significantly affect plant growth, development, reproduction and other characteristics [1]. Plant leaves are important organs for the aboveground part of the plant to obtain external resources [2,3]. As a relationship link between the plant and the external environment, leaves can respond quickly to changes in the external environment, and to a large extent, they can reflect the ecological adaptive strategy of the whole plant with greater efficiency [4,5]. Therefore, the study of leaf functional traits is highly significant for deepening our understanding of the response mechanisms of plants to environmental changes. It is also essential for predicting plant ecological adaptation and aiding in the restoration of grassland ecosystems. Among leaf functional traits, leaf dry matter content (LDMC), total nitrogen (TN) and leaf area (LA) are plastic traits that reveal different adaptive strategies for leaf resource acquisition and investment [6,7]. High LA and low LDMC indicate high efficiency in resource acquisition and utilization and low investment in leaf construction and protective tissues [8]. Low LA is advantageous in nutrient-deficient environments because it helps to reduce transpiration [5]. LDMC and LA are positively correlated with plant resource utilization strategies and are highly sensitive to climate change [9]. Plant functional groups are important units reflecting the stability and complexity of grassland ecosystems, and are useful in simplifying the processes of monitoring and modeling vegetation dynamics in more species-rich community types [10,11]. In response to natural or anthropogenic disturbances, studies focusing on plant functional groups can comprehensively evaluate how basic leaf traits and nutrients of different grassland plants adapt to environmental fluctuations [12].

Nitrogen is an important mineral obtained from the external environment for plant growth and development. In N-limited ecosystems, changes in N availability can have a significant impact on plant photosynthesis and productivity, which can rapidly trigger plants’ physiological and ecological responses [13,14]. Plant leaves play a crucial role in the global N cycle, and N inputs can alter the ability of plant leaves to produce high photosynthetic capacity and high photosynthetic N use efficiency. This is largely dependent on the plant accessing sufficient N [15,16,17]. However, excessive N inputs may lead to nutrient imbalances that can alter the functional leaf traits and adaptive capacity of plants [18]. Plants actively translocate carbon (C) and nitrogen (N) metabolites among their organs to achieve a dynamic balance of internal resource allocation, and this process is essential for maintaining the equilibrium between storage and consumption within plants [19]. Therefore, an in-depth investigation of the changes in leaf functional traits under different gradients of nitrogen addition has broad implications for the study of grassland ecology.

Grasslands constitute an essential component of terrestrial ecosystems [20,21,22]. They support a high level of species richness and contain diverse plant functional groups, rendering them a hotspot for research on plant diversity and leaf functional traits [23]. Alpine grasslands, in particular, are important ecosystems on earth, distinguished by unique climatic conditions and a rich diversity of plant species and functional groups. This offers a solid basis for studying the varying responses of different plants to nitrogen addition [24]. Typically nitrogen-limited, alpine grasslands can be employed to mimic the trend of increasing global nitrogen deposition and to examine its impacts on plant functional traits [25]. However, the marked increase in atmospheric nitrogen deposition in recent years has severely threatened plant growth, development, and reproduction [26]. Conducting in-depth studies on the sensitivity of different plant functional groups to environmental changes can further deepen our understanding of the mechanisms of species adaptation to such changes [27,28].

The Bayanbulak grassland, situated in the central part of the southern slope of the Tianshan Mountains in Xinjiang, China, is a globally representative grassland ecosystem and the first subalpine alpine steppe grassland in China. It also serves as a vital ecological barrier in Xinjiang [29]. However, few studies have investigated the impact of nitrogen addition on different functional groups in alpine ecosystems, especially using structural equation modeling (SEM) to analyze its effects on plant functional traits. Such research is critical for developing effective ecological protection and restoration strategies, as it helps elucidate the nitrogen regulation mechanism and provides a scientific basis for the rational application of nitrogen fertilizers. This study aims to (1) explore the effects of nitrogen addition on plant leaf functional traits in alpine meadows; (2) investigate how nitrogen influences the resource acquisition strategies of different functional groups; and (3) examine the linkages and influence pathways among leaf functional traits using correlation analysis and SEM. This study contributes to a comprehensive understanding of how plant functional groups adapt to alpine environments in the context of nitrogen deposition, enhances grassland productivity and sustainability, and is of great significance to the protection and restoration of alpine grassland ecosystem functions.

## 2. Materials and Methods

### 2.1. Overview of the Study Area

**Figure 1:**
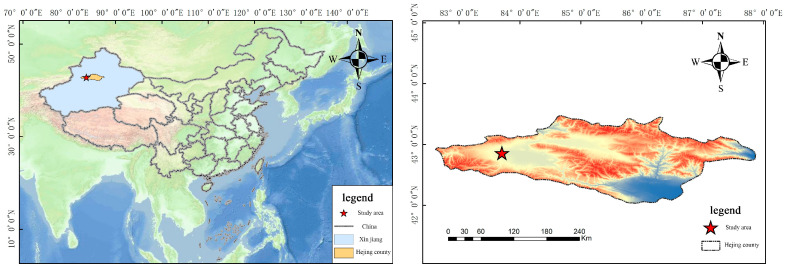
Study area.

The Bayanbulak Grassland, situated in Hejing County, Bayinguoleng Mongol Autonomous Prefecture, Xinjiang Uygur Autonomous Region, is the second-largest grassland in China. It is a high-yielding pasture in Xinjiang and a key area for research on the functional traits of grassland plant leaves [30]. This study area is located at 83°42′–85°51′ E, 42°59′–43°07′ N, with an altitude of 2000~3600 m. The mean annual temperature is −4.8 °C, with a maximum temperature of 28 °C and a minimum of −48.1 °C. The region experiences an alpine climate, with no absolute frost-free period, and the vegetation type is perennial herbaceous plants dominating alpine grassland. The mean annual precipitation is 273 mm, and the mean annual evaporation is 1250 mm. The soil type in the study area is classified as calcic cambisol. The average soil pH is 8.03, and the average concentrations of soil organic carbon, total nitrogen and total phosphorus are 34.21 g/kg, 2.67 g/kg and 0.45 g/kg, respectively. The terrain of the area is flat, the distribution of the grasses is homogeneous and the vegetation type is perennial herbaceous plants dominating alpine grassland, with the growing period concentrated in June–September. Poaceae, Rosaceae and Fabaceae are its dominant families [27] (Figure 1).

### 2.2. Experimental Design

Based on current nitrogen deposition data, historical nitrogen deposition records and the leaf functional groups and traits examined by our group, we focused on three representative functional groups and their associated traits: leaf dry matter content (LDMC), leaf area (LA), leaf width (LW), leaf length (LL), leaf circumference (LC), leaf dry weight (LDW) and leaf carbon (C), nitrogen (N) and phosphorus (P) concentrations [21,22]. The experiment was based on a randomized series of laid out sample plots. The experimental sample plots totaled 20 3 m × 3 m sample plots. Five fertilization gradients were set for nitrogen addition, and four replications were set for each treatment: control N_0_ (0 g/m^2^), low nitrogen N_5_ (5 g/m^2^), medium nitrogen N_10_ (10 g/m^2^), high nitrogen N_15_ (15 g/m^2^) and heavy nitrogen N_20_ (20 g/m^2^), respectively. A buffer strip of 0.5 m was placed between all sample plots.

The experimental sample plots were fenced and established in 2017, and the nitrogen addition treatment was started in 2021, and NH_4_NO_3_ (containing 35% nitrogen) was selected as the nitrogen additive for the simulated nitrogen addition test. The total amount of fertilizer applied to each gradient was divided into three parts, with one-third of each applied at the ends of May, June and July. The fertilizer was applied by placing pre-weighed granular fertilizer uniformly, mixed with an equal amount of water, on the soil surface of each sample plot. The control sample plot also had an equal amount of water applied to the soil surface. Fertilizer application was scheduled for cloudy days or evenings prior to rainfall to facilitate rapid dissolution into the soil. In this study, seven dominant species were selected from the plant community in the experimental plots based on their importance values from previous experiments and were categorized into three functional groups: Poaceae, Rosaceae and Fabaceae (Table 1).

### 2.3. Sample Acquisition and Processing

**Figure 2:**
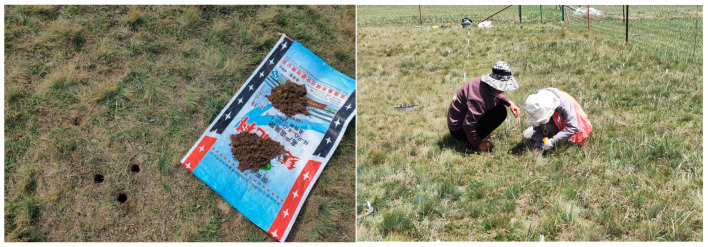
In situ photographs of plants and soils.

From 15 August to 25 August 2022, five plants in full bloom were randomly selected from each of the seven representative species in each sample plot. For each plant, 5 leaves were taken to eliminate the error of varying growth size and traits, for a total of 700 leaves sampled. Each plant was removed with a root drill and placed in a sealed bag with different sample and replicate numbers to minimize water loss and avoid leaf shriveling. Upon completion of sampling, plant samples were promptly transported to the laboratory for leaf sampling and measurement of leaf traits. The leaf functional traits assessed in this study included leaf width (LW), leaf length (LL), leaf area (LA), leaf circumference (LC), leaf dry weight (LDW), and leaf dry matter content (LDMC). The measurement of leaf width and leaf length were conducted using leaf area apparatus. Leaf dry weight was recorded in the laboratory by drying fresh leaves in an oven at 65 °C using a 1 in 10,000 balance (Figure 2). Leaf dry matter content was calculated as shown below [31]:

Leaf dry matter content (LDMC) = leaf dry weight/leaf fresh weight

At the same time, the leaves of 7 plants were brought back to the laboratory and placed in an oven to be killed first (105 °C, 30 min), dried to constant weight (65 °C, 48 h) for weighing (precision of 0.01 g) and then ground and sieved through a 2 mm sieve to determine the nutrient content, including the TOC, TN and TP of the leaves. Plant nutrient content was determined by grinding the plant samples into a 100-mesh sieve, and the nutrient content of each functional group was measured four times at each gradient. Plant organic carbon was determined by the K_2_Cr_2_O_7_-FeSO_4_ titration method, plant total nitrogen was determined by the semi-micro Kjeldahl method and plant total phosphorus was determined by the HClO_4_-H_2_SO_4_ molybdenum and antimony colorimetric method.

### 2.4. Data Processing

Data were summarized and organized using Excel 2010. Statistical analyses were conducted with SPSS 27.0, and graphical representations were generated using R 4.3.2. Specifically, the R packages corrplot, ggplot2 and piecewiseSEM were employed for data analysis and visualization. One-way analysis of variance (ANOVA) was utilized to assess the effects of nitrogen addition on plant leaf dry matter content, plant stoichiometric properties and leaf functional traits. Structural equation modeling (SEM) was applied to elucidate the pathways through which plant dry matter content responds to nitrogen addition. Firstly, Pearson correlation analysis was used to test the correlation between plant leaf functional traits. Secondly, three plant-related significant factors were screened out, and structural equation modeling was used to explore the significant response pathways of the dry matter content of the three plants regarding nitrogen addition. Based on the concept of structural equation modeling, when *p* > 0.05, if there are path coefficients >1, they need to be deleted until all the path coefficients are less than 1. The smaller the Fisher’s, AIC and BIC values, the better fitted the structural equation model.

## 3. Results

### 3.1. Response of Leaf Functional Traits to Nitrogen Addition

This study analyzed leaf functional traits and their relationships with nutrient cycling, interspecific competition, and adaptive strategies across different nitrogen addition gradients in Poaceae, Fabaceae and Rosaceae. Using R and SPSS for statistical analysis, one-way ANOVA revealed significant changes in leaf functional traits under varying nitrogen gradients. The most significant changes were observed in leaf area (LA) and leaf length (LL) within Poaceae (*p* < 0.05). Specifically, Fabaceae reached maximum values of 0.87 cm^2^ and 2.38 cm for LA and LL, respectively, under the N_15_ gradient, while they exhibited minimum values of 0.52 cm^2^ and 1.71 cm under the N_10_ gradient. Poaceae plants reached maximum values of 0.64 cm^2^, 4.79 cm and 15.87 cm for LA, LL and LC, respectively, under the N_15_ gradient, but their LA and LL varied significantly (*p* < 0.05) under the N_10_ gradient. With the N_10_ gradient, their LL and leaf perimeter decreased to the minimum. Meanwhile, the LA, LC and LDW of Rosaceae maintained high levels under different N addition gradients, but their variations were not significant (*p* > 0.05), showing the stability of this plant in N resource utilization. The trend of leaf dry weight (LDW) changes was consistent in Poaceae and Fabaceae, with no significant differences observed under varying nitrogen gradients (*p* > 0.05). This suggests that these functional groups may share similar mechanisms for nitrogen resource utilization.

**Figure 3:**
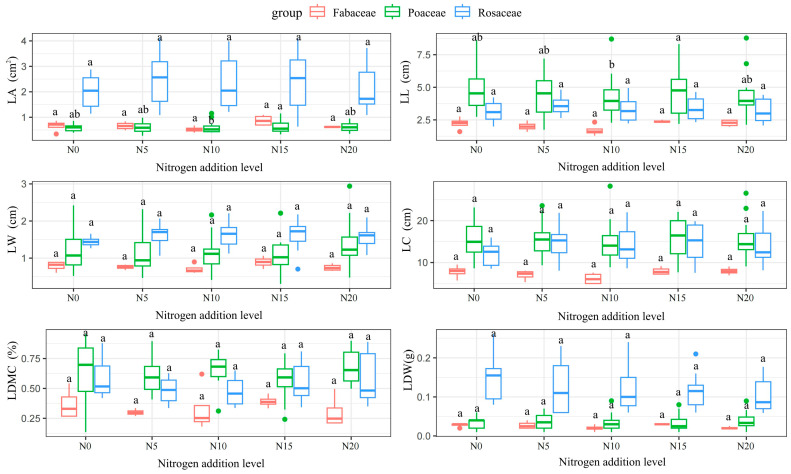
Functional traits of the leaf blade in response to nitrogen addition. LA: leaf area; LL: leaf length; LW: leaf width; LC: leaf circumference; LDMC: leaf dry matter content; LDW: leaf dry weight. Note: Different lowercase letters indicate significant differences between treatments (*p* < 0.05).

Regarding LDMC, Poaceae, Rosaceae and Fabaceae exhibited non-significant changes under different nitrogen addition gradients. However, each functional group displayed distinct patterns of variation. The LDMC of Poaceae plants reached a maximum value of 67% at N_10_ and N_20_ gradients, while it decreased to a minimum of 57% at the N_15_ gradient. The LDMC of Rosaceae had the highest value of 0.59% under the control N_0_ treatment, while it generally decreased under nitrogen addition conditions and showed a tendency of decreasing and then increasing with increasing gradients of nitrogen addition, including 0.58% in the N_20_ treatment. The LDMC of Fabaceae as a whole was significantly lower than that of Fabaceae and Rosaceae, and the trend was not consistent with the former two, with the LDMC reaching a maximum value of 0.39% under the N_15_ treatment and decreasing to 0.30% under the N_5_ and N_20_ gradients. Changes in these plant functional traits reflected the efficiency of each functional group in utilizing nitrogen resources and revealed the complex relationship with nutrient cycling, interspecific competition and adaptation strategies [32]. It is hypothesized that plants with higher leaf dry matter content may enhance their nitrogen acquisition capacity, thereby increasing their competitive advantage, promoting nutrient cycling and accelerating ecosystem productivity and stability [33] (Figure 3).

### 3.2. Response of Plant Nutrient Content to Nitrogen Addition

**Figure 4:**
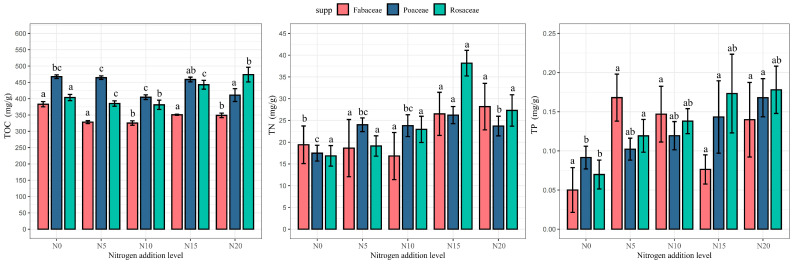
Plant stoichiometry in response to nitrogen addition. TOC: leaf total organic carbon; TN: leaf total nitrogen; TP: leaf total phosphorus. Note: Mean ± standard error. Different lowercase letters indicate significant differences between treatments (*p* < 0.05).

In this study, the effects of nitrogen addition on the nutrient content of three plant functional groups, Fabaceae, Rosaceae and Fabaceae, were investigated by univariate analysis. The results showed that the nutrient indexes of all three plant species changed significantly (*p* < 0.05) with increasing gradients of nitrogen addition, thereby reflecting the importance of nitrogen in nutrient cycling. The TOC of Fabaceae decreased significantly with increasing nitrogen concentration to 404.51 mg/g at N_10_, and the TP increased to 0.17 mg/g at N_20_, indicating that their TOC may decrease in high nitrogen environments due to the uneven distribution of resources. Meanwhile, the TN reached 26.22 mg/g at the N_15_ treatment level, indicating an increase in resource utilization efficiency. The TOC of Rosaceae exhibited a trend of first decreasing and then increasing, with a minimum value of 381.21 mg/g at the N_10_ treatment, increasing to 473.8 mg/g at the N_20_ treatment. Under low nitrogen conditions, plants competed for resources by enhancing root growth, which led to a decrease in TOC. As nitrogen concentration increased, plant growth potential and photosynthesis were enhanced, resulting in the maximum TOC at the N_20_ treatment [30]. The level of TN and TP increased gradually with nitrogen addition, reaching a minimum value of 16.86 mg/g and 0.07 mg/g at control N_0_ and a maximum value of 38.17 mg/g and 0.18 mg/g at N_15_ and N_20_, respectively. This indicates elevated nutrient uptake for enhanced competitiveness. The TP of Fabaceae increased with N addition and reached a maximum value of 0.17 mg/g at the N_5_ treatment, while TP content at N_15_ was similar to that of N_0_, which fully indicated their effective utilization of nutrient resources under suitable N conditions. At the N_10_ treatment, TOC and TN reached their minimum values of 325.36 mg/g and 16.81 mg/g, respectively, indicating nutrient competition pressure. In contrast, the control group exhibited the maximum TOC at 382.95 mg/g, while TN was highest at the N_20_ treatment, reaching 28.17 mg/g. These results reflect the adaptive strategies of Fabaceae under varying nitrogen conditions and highlight the significance of nutrient cycling (Figure 4).

### 3.3. Correlation Analysis of Leaf Functional Traits and Plant Nutrient Content

**Figure 5:**
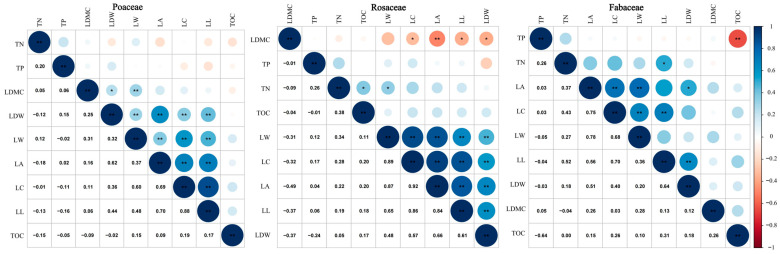
Heat map of correlation between leaf functional traits and plant nutrient content. The half-matrix plot in the figure shows Pearson’s correlation coefficients with a color gradient, ** indicates that the correlation is significant at the 0.01 level and * indicates that the correlation is significant at the 0.05 level.

To examine the correlation between leaf functional traits and plant nutrient content across the three plant functional groups, Pearson correlation analysis was employed. No significant correlation was detected among TOC, TN and TP in Poaceae plants (Figure 5). However, LW, LA, LC, LL and LDW exhibited significant positive correlations, with correlation coefficients of 0.32, 0.62, 0.36 and 0.44, respectively. Meanwhile, LDMC also showed a significant correlation with LDW and LW, with the correlation values of 0.26 and 0.31, respectively. This suggests that under similar environmental conditions, Poaceae plants enhance photosynthetic efficiency and water use by optimizing leaf functional traits to enhance survival competitiveness. There was a significant correlation between TN and TOC and LW in Rosaceae, with correlation values of 0.38 and 0.34, respectively. There was also a significant positive correlation between LC, LL, LW and LDW, with correlation coefficients of 0.57, 0.61 and 0.48, respectively. This indicates that Rosaceae may employ specific nutrient cycling mechanisms in resource acquisition and utilization, supporting efficient nutrient uptake and partitioning through adaptive strategies and potentially prioritizing certain nutrient sources. In summary, the correlation between leaf functional traits and plant nutrient content was not significant for Poaceae plants under different N addition gradients, but TN and LW for Rosaceae and TN and LL for Fabaceae had significant correlations, and TN and TOC for Rosaceae and TP and TOC for Fabaceae showed opposite significant correlations.

### 3.4. Pathway Analysis of the Effects of Changes in the Dry Matter Content of Leaf Blades

**Figure 6:**
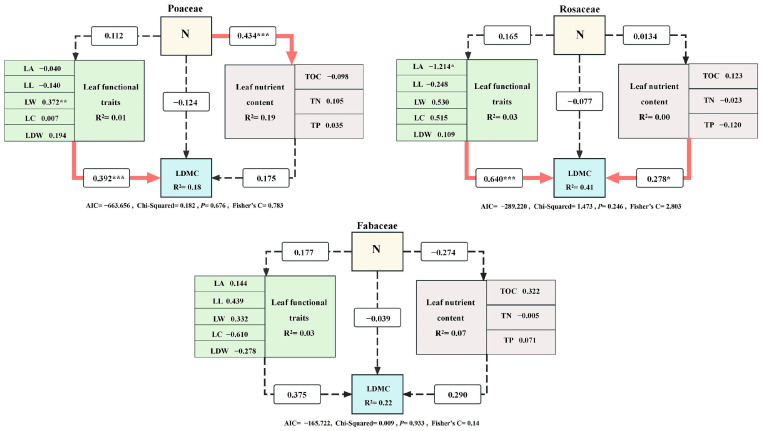
Structural equation modeling of the three plant species. Red arrows and dashed crosses indicate significant and non-significant correlations, respectively, and the significance of path coefficients on the arrows is indicated by the level of significance: *** *p* < 0.001, ** *p* < 0.01, * *p* < 0.05.

To elucidate the pathways through which plant dry matter content responds to nitrogen addition, this study employed SEM to explore the significant pathways of dry matter content in three plant functional groups. In Fabaceae, SEM revealed that nitrogen addition influenced leaf functional traits, leaf nutrient content and leaf dry matter content through path coefficients of 0.112, 0.434 and −0.124, respectively. Nitrogen addition significantly and directly influences leaf nutrient content in Poaceae, with a path coefficient of 0.434. Additionally, plant functional traits directly affect leaf dry matter content, with a path coefficient of 0.392. The path coefficients of nitrogen addition on leaf functionality, leaf nutrient content and leaf dry matter content in Rosaceae were 0.165, 0.0134 and 0.077, respectively. Leaf nutrient content and plant functional traits in Rosaceae directly and significantly affected leaf dry matter content, with path coefficients of 0.640 and 0.278, respectively. The SEM results of Fabaceae plants showed that nitrogen addition significantly affected leaf nutrient content, while no significant effect was observed between plant functional traits and leaf dry matter content, with path coefficients of 0.177, −0.274 and −0.039, respectively. Fabaceae may possess a stronger ability to regulate resources and maintain a relatively stable growth strategy in nitrogen-supply-poor or abundant environments through mechanisms such as nitrogen fixation by rhizobacteria [34]. Among the three plant functional groups, Rosaceae had the highest R of 0.41 for dry matter content interpretation, followed by Fabaceae, with an R of 0.22, while Fabaceae had the lowest R of 0.18 (Figure 6).

## 4. Discussion

### 4.1. Effect of Nitrogen Addition on Leaf Functional Traits

Plants adapt to external environmental changes by regulating leaf functional traits, and nitrogen is one of their major limiting elements. Moderate nitrogen addition can alleviate the negative effects of drought on plants, and different plants show significant differences in their strategies for environmental adaptation [35,36,37]. In the present study, we found that LA, LL and LDMC of Poaceae plants varied significantly, exhibited strong plasticity, and were more capable of adapting to exogenous nitrogen, which was basically consistent with Yu’s study [38]. The differences in LDMC among the three functional groups were not significant. In Fabaceae, the changes in LDMC under nitrogen addition gradients were less pronounced than those in Rosaceae. This can be attributed to the unique nitrogen fixation capacity, growth strategy and ecological adaptations of Fabaceae [39,40]. The significant changes in LA and LL observed in Poaceae and Rosaceae, but not in Fabaceae, under different nitrogen addition gradients may reflect specific responses of plant functional groups to nitrogen availability. It has been hypothesized that soil pH, when either too low or too high, may lead to nutrient deficiencies, which can in turn affect functional traits such as leaf thickness and chlorophyll content [41]. Water, conversely, is directly related to plant transpiration and photosynthesis [42]. In water-deficient environments, leaves may exhibit wilting and yellowing at the edges, thereby compromising the overall health and growth of the plant and consequently influencing its leaf traits. Nitrogen addition can alter the leaf morphology of grass plants by increasing leaf area and thickness and enhancing photosynthesis. However, excessive nitrogen fertilization can result in plant fragility and environmental issues [43].

### 4.2. Effect of Nitrogen Addition on Plant Nutrient Content

Carbon (C) acts as an energy source and substrate for plant physiological and biochemical processes, while nitrogen (N) and phosphorus are major limiting factors for plant growth in terrestrial ecosystems [44]. Studies have shown that stable C and N in grassland ecosystems can reflect the consistency of ecophysiological processes within these ecosystems [45]. In this study, the total nitrogen (TN) content of Poaceae, Rosaceae and Fabaceae increased significantly with increasing nitrogen addition gradients, indicating an enhanced uptake of exogenous nitrogen by these plants. This phenomenon has important implications for long-term nutrient cycling and ecosystem functioning, as nitrogen accumulation promotes plant growth and increases ecosystem primary productivity. However, the limiting effect of phosphorus is subsequently exacerbated, as evidenced by the significant increase in total phosphorus (TP). Although plants are able to access more nitrogen, the relative scarcity of phosphorus may constrain their growth and lead to trophic imbalances, which can affect overall competitiveness and ecological response [46]. Nitrogen addition has been found to decrease C and increase N, suggesting that exogenous N addition weakens plant N limitation and increases plant growth rates [47]. TOC content was significantly reduced in Poaceae and Fabaceae, whereas it was significantly increased in Rosaceae. This may be related to the ability of different plant functional groups to regulate non-structural and structural carbohydrates [48]. Exogenous N addition altered the mode of carbohydrate accumulation in Fabaceae, leading to a decrease in TOC. In contrast, Rosaceae increased TOC through enhanced photosynthetic efficiency and adaptive strategies. These findings indicate that while nitrogen addition promotes plant growth, it also exerts competitive pressure on other nutrients, particularly phosphorus [49].

### 4.3. Drivers of Nitrogen Addition on Plant Functional Traits

Nitrogen addition can promote rapid plant growth and intensify competition among conspecifics. This study found that nitrogen addition altered plant functional traits and resource allocation, thereby stabilizing the relative positions of species and community structure [17]. Experiments involving multiple levels of nitrogen addition in temperate grasslands and alpine steppes have demonstrated that nitrogen addition induces alterations in plant leaf morphology and physiological processes, thereby influencing nutrient absorption capabilities [50,51]. Nutrient addition experiments also showed a significant decrease in grassland plant diversity due to differences in plant response to nutrients in different functional groups [47,52,53]. In this study, no significant correlation was detected between leaf nutrient content in Poaceae. However, TN in Fabaceae and Rosaceae was significantly correlated with LL and LW, indicating a relationship between their growth strategies and nutrient acquisition optimization. Trait correlations in plants are essential for understanding adaptive and ecological functions. The lack of significant correlations between TOC, TN and TP in Poaceae plants may be attributed to functional redundancy [54]. In addition, factors such as moisture, light and soil properties may mask potential correlations among traits, and plants also need to trade off growth and reproduction in resource-limited environments, which can lead to variability in certain traits [55]. In conclusion, negative correlations in Fabaceae are closely related to physiological limitations and competitive nutrient uptake, which may cause a prioritization of the uptake of key nutrients under nutrient deficiencies or environmental stresses and adjust the uptake ratios of different nutrients to adapt to growth requirements [56].

Previous studies have demonstrated that nitrogen addition significantly impacts plant functional traits in most cases [27,57,58]. SEM results indicated that nitrogen addition influenced LDMC differently across various plant groups. Rosaceae exhibited the highest explanatory power, with an R^2^ value of 0.41, whereas Fabaceae had the lowest, at R^2^ = 0.18. This discrepancy reflects the varying efficiencies of nitrogen uptake and utilization between these two plant groups. The high explanatory power of Rosaceae stems from special leaf structures (e.g., high leaf area), superior N uptake efficiency and the ability to adapt to the environment by regulating stress tolerance and water use efficiency. The synergistic effect between these characteristics enhances their ecological adaptability [59]. The SEM of Fabaceae showed no significant effect of N addition on their traits or dry matter content. Fabaceae have the ability to biologically fix nitrogen through a symbiotic relationship with rhizobacteria and are able to grow in nitrogen-poor soils, indicating their independence from external nitrogen sources [39]. Studies have shown that nitrogen addition does not significantly affect their growth and dry matter mass, indicating that they preferentially rely on nitrogen fixation mechanisms and possess adaptive strategies [56]. These traits confer a unique ecological position while enhancing soil quality. Nitrogen addition has a significant and direct impact on Rosaceae, primarily due to their high leaf nutrient content, robust root development and ability to adapt to diverse ecosystems. These characteristics enable them to efficiently absorb and utilize nitrogen [60]. Comparatively, Poaceae plants have a slower N response due to their simple leaf structure and lower root uptake capacity, and thus have a more indirect effect. Fabaceae, on the other hand, rely on rhizobia for biological nitrogen fixation and have a low external nitrogen demand, which makes them less responsive to nitrogen addition as well. Thus, the growth of Rosaceae was significantly affected by nitrogen, whereas Poaceae and Fabaceae showed an indirect response.

## 5. Conclusions

This study investigated the effects of nitrogen addition on alpine grassland plants using a combination of community surveys, indoor experiments, correlation analysis and structural equation modeling. Poaceae plants exhibited significant differences in leaf area and leaf length across different nitrogen addition gradients, while nutrient contents in Rosaceae and Fabaceae showed substantial changes. Pearson’s correlation analysis revealed that the functional traits of Poaceae were less correlated with nutrient contents in Fabaceae under varying nitrogen addition gradients. In contrast, significant positive and negative correlations were observed between Rosaceae and Fabaceae. Structural equation modeling indicated that nitrogen addition directly affected carbon (C), nitrogen (N) and phosphorus (P) contents. Leaf dry matter content (LDMC) in Poaceae was influenced by leaf width, while LDMC in Rosaceae was affected by leaf area and nutrient contents. These factors were also significant in Fabaceae. Poaceae demonstrated greater plasticity, with significant changes in leaf area, leaf length and LDMC, indicating stronger adaptability to exogenous nitrogen. The response of Fabaceae to nitrogen addition was weaker due to their nitrogen-fixing capacity, whereas Rosaceae exhibited significant growth responses through efficient nitrogen uptake and adaptive strategies. Overall, nitrogen addition promoted plant growth but also intensified competitive pressure for other nutrients, particularly phosphorus. This finding not only deepens our understanding of plant leaf functional traits and their interactions with C, N and P in the Bayanbulak alpine grassland, but also provides new insights for predicting the impacts of future climate change on alpine grassland ecosystems.

## Figures and Tables

**Table 1:** List of plant species.

Family	Species	Abbreviations
Poaceae	*Festuca ovina* L.	*Fo*
	*Agropyron cristatum* (Linn.) Gaertn.	*Ac*
	*Koeleria cristata* (L.) Pers.	*Kc*
	*Poa pratensis* L.	*Pp*
Rosaceae	*Potentilla fragarioides* L.	*Pf*
	*Potentilla bifurca* Linn.	*Pb*
Fabaceae	*Astragalus adsurgens* (Fisch.) Bunge.	*Aa*

## Data Availability

The data presented in this study are available on request from the corresponding author.

## References

[1] Wang Z., Zhang J., Li Z., Liu H., Wang L., Wang W., Wang Y., Liang C. (2021). Single grazing is more detrimental to grasslands than mixed grazing: Evidence from the response of functional traits of dominant plants to grazing systems. Front. Ecol. Evol..

[2] Moeneclaey I., Schelfhout S., Blondeel H., Van Coillie F., Verheyen K., Baeten L. (2024). Leaf trait variation in grassland plant species in response to soil phosphorus. J. Veg. Sci..

[3] Louarn G., Chabbi A., Gastal F. (2020). Nitrogen concentration in the upper leaves of the canopy is a reliable indicator of plant N nutrition in both pure and mixed grassland swards. Grass Forage Sci..

[4] Walker T.W., Alexander J.M., Allard P.M., Baines O., Baldy V., Bardgett R.D., Capdevila P., Coley P.D., David B., Defossez E. (2022). Functional Traits 2.0: The power of the metabolome for ecology. J. Ecol..

[5] Wang J., Wang X., Ji Y., Gao J. (2022). Climate factors determine the utilization strategy of forest plant resources at large scales. Front. Plant Sci..

[6] Poorter L., Rozendaal D.M.A. (2008). Leaf size and leaf display of thirty-eight tropical tree species. Oecologia.

[7] Wright I.J., Reich P.B., Westoby M., Ackerly D.D., Baruch Z., Bongers F., Cavender-Bares J., Chapin T., Cornelissen J.H.C., Diemer M. (2004). The worldwide leaf economics spectrum. Nature.

[8] Akram M.A., Zhang Y., Wang X., Shrestha N., Malik K., Khan I., Ma W., Sun Y., Li F., Ran J. (2022). Phylogenetic inde-pendence in the variations in leaf functional traits among different plant life forms in an arid environment. J. Plant Physiol..

[9] Reich P.B., Ellsworth D.S., Walters M.B., Vose J.M., Gresham C., Volin J.C., Bowman W.D. (1999). Generality of leaf trait relationships: A test across six biomes. Ecology.

[10] Funk J.L., Larson J.E., Ames G.M., Butterfield B.J., Cavender-Bares J., Firn J., Laughlin D.C., Sutton-Grier A.E., Williams L., Wright J. (2017). Revisiting the Holy Grail: Using plant functional traits to understand ecological processes. Biol. Rev..

[11] Jiang L., Wan S., Li L. (2009). Species diversity and productivity: Why do results of diversity-manipulation experiments differ from natural patterns?. J. Ecol..

[12] Cui E., Weng E., Yan E., Xia J. (2020). Robust leaf trait relationships across species under global environmental changes. Nat. Commun..

[13] Niu D., Yuan X., Cease A.J., Wen H., Zhang C., Fu H., Elser J.J. (2018). The impact of nitrogen enrichment on grassland ecosystem stability depends on nitrogen addition level. Sci. Total Environ..

[14] Yuan X., Niu D., Gherardi L.A., Liu Y., Wang Y., Elser J.J., Fu H. (2019). Linkages of stoichiometric imbalances to soil microbial respiration with increasing nitrogen addition: Evidence from a long-term grassland experiment. Soil Biol. Biochem..

[15] Chapin F.S., Schulze E.D., Mooney H.A. (1990). The ecology and economics of storage in plants. Annu. Rev. Ecol. Syst..

[16] Reich P.B., Walters M.B., Ellsworth D.S. (1992). Leaf life-span in relation to leaf, plant, and stand characteristics among diverse ecosystems. Ecol. Monogr..

[17] Wang B., Gong J., Zhang Z., Yang B., Liu M., Zhu C., Shi J., Zhang W., Yue K. (2019). Nitrogen addition alters photosynthetic carbon fixation, allocation of photoassimilates, and carbon partitioning of Leymus chinensis in a temperate grassland of Inner Mongolia. Agric. For. Meteorol..

[18] Mao Q., Lu X., Mo H., Gundersen P., Mo J. (2018). Effects of simulated N deposition on foliar nutrient status, N metabolism and photosynthetic capacity of three dominant understory plant species in a mature tropical forest. Sci. Total. Environ..

[19] Furze M.E. (2019). Understanding Whole-Plant Nonstructural Carbohydrate Storage in a Changing World. Ph.D. Dissertation.

[20] Wang J., Liu J.J., Liu C., Ding X.Y., Wang Y. (2023). Species niche and interspecific associations alter flora structure along a ferti-lization gradient in an alpine meadow of Tianshan Mountain, Xinjiang. Ecol. Indic..

[21] Wang J., Liu J., Liu C., Ding X., Ma Y., Yang J., Xu Z. (2024). Effects of short-term nitrogen addition on the recovery of alpine grassland in the Tianshan Mountains of Xinjiang, China. Ecol. Evol..

[22] Liu C., Liu J., Wang J., Ding X. (2024). Effects of Short-Term Nitrogen Additions on Biomass and Soil Phytochemical Cycling in Alpine Grasslands of Tianshan, China. Plants.

[23] Tuo H., Ghanizadeh H., Ji X., Yang M., Wang Z., Huang J., Wang Y., Tian H., Ye F., Li W. (2024). Moderate grazing enhances ecosystem multifunctionality through leaf traits and taxonomic diversity in long-term fenced grasslands. Sci. Total. Environ..

[24] Xiang X., De K.J., Lin W., Feng T., Li F., Wei X. (2024). The ecological niche characteristics and interspecific associations of plant species in the alpine meadow of the Tibetan Plateau affected plant species diversity under nitrogen addition. PeerJ.

[25] Wang F., Shi G., Nicholas O., Yao B., Ji M., Wang W., Ma Z., Zhou H., Zhao X. (2018). Ecosystem nitrogen retention is regulated by plant community trait interactions with nutrient status in an alpine meadow. J. Ecol..

[26] Bobbink R., Hicks K., Galloway J., Spranger T., Alkemade R., Ashmore M., Bustamante S., Cinderby S., Davidson E., Dentener F. (2010). Global assessment of ni-trogen deposition effects on terrestrial plant diversity: A synthesis. Ecol. Appl..

[27] Cortois R., Schröder-Georgi T., Weigelt A., van der Putten W.H., De Deyn G.B. (2016). Plant–soil feedbacks: Role of plant functional group and plant traits. J. Ecol..

[28] McLaren J.R., Turkington R. (2010). Ecosystem properties determined by plant functional group identity. J. Ecol..

[29] Xu X., Wang X., Jia H.T., Zhu X.P. (2020). Seasonal landscape pattern changes in Bayanbulak swan lake alpine wetland. J. Agric. Resour. Environ..

[30] Liang Y.Y., Zhang L.X., Zhou X.L., Fan L.L., Mao J.F., Li Y.M. (2023). Response of soil aggregate structure and nutrient content to simulated nitrogen and phosphorus deposition in alpine grassland of Tianshan Mountain. J. Ecol..

[31] Pérez-Harguindeguy N., Díaz S., Garnier E., Lavorel S., Poorter H., Jaureguiberry P., Bret-Harte M.S., Cornwell W.K., Craine J.M., Gurvich D.E. (2013). New handbook for standardised measurement of plant functional traits worldwide. Aust. J. Bot..

[32] Shi N., Wang J., Zhang L., Wu Y. (2024). Divergent adaptive strategies of paired Meconopsis species and their impact factors along the elevational gradients in the south-eastern margin of Qinghai-Tibet Plateau. J. Mt. Sci..

[33] Happonen K., Virkkala A., Kemppinen J., Niittynen P., Luoto M. (2022). Relationships between above-ground plant traits and carbon cycling in tundra plant communities. J. Ecol..

[34] Yang J., Lan L., Jin Y., Yu N., Wang D., Wang E. (2021). Mechanisms underlying legume–rhizobium symbioses. J. Integr. Plant Biol..

[35] Singh H., Kumar N., Kumar A. (2024). Enhancing Resource Use Efficiency in Crops Through Plant Functional Traits. Plant Functional Traits for Improving Productivity.

[36] Ohyama T. (2010). Nitrogen as a major essential element of plants. Nitrogen Assimilation in Plants.

[37] Luo Y., Du L., Zhang J., Ren H., Shen Y., Zhang J., Li N., Tian R., Wang S., Liu H. (2024). Nitrogen addition alleviates the adverse effects of drought on plant productivity in a temperate steppe. Ecol. Appl..

[38] Yu H.-W., He W.-M. (2017). Negative legacy effects of rainfall and nitrogen amendment on leaf lifespan of steppe species. J. Plant Ecol..

[39] Tang J., Li W., Wei T., Huang R., Zeng Z. (2024). Patterns and Mechanisms of Legume Responses to Nitrogen Enrichment: A Global Meta-Analysis. Plants.

[40] Li X., Li Y., Shen H., Li S., Zhao Z., Xiao J., Zhang R., Shi H., Zuo H., Danjia T. (2024). Different responses of individuals, functional groups and plant communities in CSR strategies to nitrogen deposition in high-altitude grasslands. Sci. Total. Environ..

[41] Yu J., Li Q., Wu X., Zhu C., Huang S., Yang F., Hou X. (2023). Adaptational responses of leaf functional traits of Dicranopteris dichotoma to environmental factors in different vegetational restoration stages. Glob. Ecol. Conserv..

[42] Rawson H.M., Begg J.E., Woodward R.G. (1977). The effect of atmospheric humidity on photosynthesis, transpiration and water use efficiency of leaves of several plant species. Planta.

[43] Wang D., Maughan M.W., Sun J., Feng X., Miguez F., Lee D., Dietze M.C. (2012). Impact of nitrogen allocation on growth and photosynthesis of Miscanthus (*Miscanthus × giganteus*). GCB Bioenergy.

[44] Pandey N. (2018). Role of plant nutrients in plant growth and physiology. Plant Nutrients and Abiotic Stress Tolerance.

[45] Klumpp K. (2021). Carbon, nitrogen and phosphorus cycling in cropland and grassland ecosystems. Agronomy.

[46] Gatiboni L., Brunetto G., Pavinato P.S., George T.S. (2020). Editorial: Legacy phosphorus in agriculture: Role of past management and perspectives for the future. Front. Earth Sci..

[47] Glass A.D. (2003). Nitrogen use efficiency of crop plants: Physiological constraints upon nitrogen absorption. Crit. Rev. Plant Sci..

[48] Li Z., Yu X., Jia G. (2022). The anatomical structure of woody plants in arid habitats is closely related to nonstructural carbohydrates storage. For. Ecosyst..

[49] Su Y., Dong K., Wang C., Liu X. (2024). A meta-analysis of the impacts of nitrogen addition on plant multiple-element contents in natural ecosystems. Plant Ecol..

[50] Zhou H., Xu X., Jiang X., Ding B., Wu P., Ding F. (2022). Plant functional trait responses to dolomite and limestone karst forests in Southwest China. Forests.

[51] Zhou M., Yang Q., Zhang H., Yao X., Zeng W., Wang W. (2020). Plant community temporal stability in response to nitrogen addition among different degraded grasslands. Sci. Total. Environ..

[52] Chen Y., Han W., Tang L., Tang Z., Fang J. (2013). Leaf nitrogen and phosphorus concentrations of woody plants differ in re-sponses to climate, soil and plant growth form. Ecography.

[53] Su Y., Ma X., Gong Y., Li K., Han W., Liu X. (2021). Responses and drivers of leaf nutrients and resorption to nitrogen enrichment across northern China’s grasslands: A meta-analysis. CATENA.

[54] Xiao H., Li P., Monaco T.A., Liu Y., Rong Y. (2023). Nitrogen and phosphorus additions alter foliar nutrient concentrations of dominant grass species and regulate primary productivity in an Inner Mongolian meadow steppe. Sci. Total. Environ..

[55] Wang C., Lü W.W., Sun J.P., Zhou Y., Jiang L.L., Li B.W., Zhang S.R., Xia L., Wang Q., Que D.S. (2021). Responses of plant leaf traits to simulated rainfall changes in alpine region. Acta Ecol. Sin..

[56] Ri X., Dai D., Xu X. (2021). The symbiotic nitrogen fixation by legumes in a legume-companion and a legume-dominant alpine steppe on the central Tibetan Plateau. Ecol. Res..

[57] Wu W., Sun R., Zhao G., Zheng Z., He Y., Liu L., Zhou G., Zhang Y., Xu Z. (2024). Climate shifts biomass allocation by altering plant functional group in alpine vs. temperate grasslands on both Inner Mongolian and Tibetan plateaus. CATENA.

[58] Liu C., Liu Y., Guo K., Qiao X., Zhao H., Wang S., Zhang L., Cai X. (2018). Effects of nitrogen, phosphorus and potassium addition on the productivity of a karst grassland: Plant functional group and community perspectives. Ecol. Eng..

[59] Valverde-Barrantes O.J., Smemo K.A., Blackwood C.B. (2015). Fine root morphology is phylogenetically structured, but nitrogen is related to the plant economics spectrum in temperate trees. Funct. Ecol..

[60] Cai J., Fu J., Liu H., Li T., Feng X., Lu J., Wang R., Jiang Y. (2023). Divergent responses of leaf mineral nutrient concentrations among plant families and functional groups to nitrogen addition and irrigation in a semi-arid grassland. Plant Soil.

